# Are There Changes in the Nursing Managers’ Expectations of the Professional Quality of New Graduate Nurses after the Emerging of Infectious Disease Pandemic?

**DOI:** 10.31662/jmaj.2023-0015

**Published:** 2023-09-13

**Authors:** Michihiro Tsubaki, Jun Kako, Yuji Koga, Masamitsu Kobayashi, Yoji Endo, Yasutaka Kimura, Hana Kiyohara, Michiko Ishida, Yoko Nishida, Kimie Harada, Kohei Kajiwara, Yoshiyasu Ito, Yuki Wakiguchi, Shinsuke Sasaki, Seiji Hamanishi, Makoto Yamanaka, Takahiro Kakeda

**Affiliations:** 1 School of Nursing, Kitasato University, Sagamihara, Japan; 2 College of Nursing Art and Science, University of Hyogo, Akashi, Japan; 3 Faculty of Nursing, Kawasaki University of Medical Welfare, Kurashiki, Japan; 4 Graduate of Nursing Science, St. Luke’s International University, Tokyo, Japan; 5 Faculty of Nursing, Kansai University of Social Welfare, Ako, Japan; 6 Department of Nursing, Meio University, Nago, Japan; 7 Faculty of Nursing, Japanese Red Cross Kyushu International College of Nursing, Munakata, Japan; 8 Department of Nursing Science, Okayama Prefectural University, Soja, Japan; 9 School of Nursing, Aichi Medical University, Nagakute, Japan; 10 Faculty of Nursing, Kawasaki City College of Nursing, Kawasaki, Japan

**Keywords:** emerging infectious disease, management, new graduate nurses, nurse managers, professional quality

The coronavirus disease (COVID-19) pandemic, a public health emergency of international concern, has had a major impact on hospital nurses. Nurses’ mental health during the COVID-19 pandemic is a common issue in the international healthcare system ^(1)^. Additionally, it also affects the competencies nurses are expected to possess ^(2)^. In sustainable nursing management, the characteristics required by future nurses are considered an important point of view ^(3)^. It has been reported that mental stress during the COVID-19 pandemic affects the recruitment of new graduate nurses. However, the types of changes observed in the recruitment of new graduate nurses by nursing managers remain unclear. Therefore, this research aimed to clarify the qualities that nursing managers expect of new graduate nurses in the post-coronavirus world.

This study adopted a qualitative and descriptive research design to clarify the professional qualities nursing managers expect of new graduate nurses in the post-coronavirus world. Interviews were conducted with participants sitting on a nurse recruitment examination during the COVID-19 pandemic from March 31, 2021, to August 17, 2021. The newly graduated nurses in this study had completed basic nursing education in four-year universities and were beginning their careers in the medical field for the first time. To select participants strongly affected by the COVID-19 pandemic, the study focused on those who worked in medical institutions in areas where a state of emergency had been declared.

The survey questionnaire included items related to the professional qualities that nursing managers expect of new graduate nurses in the post-coronavirus world. Semi-structured and based on a researcher-generated interview guide, the interviews were conducted online according to the participants’ preferences as a COVID-19 prevention measure. The interviews were recorded using an Integrated Circuit recorder and transcribed verbatim with the participants’ permission. For data analysis, we carefully read verbatim and extracted descriptions of the human qualities that nursing managers expect of newly graduated nurses. We categorized them into those who expected new qualities in new graduate nurses due to COVID-19 and those who did not.

Written informed consent for participation was obtained from all subjects after they were informed verbally and in writing that participation was entirely voluntary, and they could refuse participation or withdraw early without consequences. The privacy and anonymity of all participants were protected, and ample consideration was given to their psychological and physical state when seeking signed consent. This study was approved by the Institutional Review Board of the Research Ethics Committee of the College of Nursing Art and Science and the Research Institute of Nursing Care for People and Community, University of Hyogo, Japan (approval no. 2020F29).

Interviews were conducted with fifteen participants whose job titles were “Director of nursing,” “Assistant director of nursing,” and “Nursing department staff,” all of which correspond to nursing managers. The details of the hospital facilities in areas affected by COVID-19 are presented in Table 1.

**Table 1.** Basic Attributes of Participants and Expected Changes in the Professional Qualities of New Graduate Nurses after COVID-19.

Job title		Affiliation facility		Responses^※※^	
		Number of infected^※^	Number of beds	Q1	Q2
A	Assistant director of nursing	More than 200	>1000	No	No
B	Nursing department staff	More than 200	500-999	No	No
C	Assistant director of nursing	More than 200	100-499	Unknown	Unknown
D	Nursing department staff	More than 200	500-999	No	No
E	Director of nursing	More than 200	500-999	Yes	Yes
F	Director of nursing	120-200	>1000	Yes	No
G	Assistant director of nursing	More than 200	>1000	No	Yes
H	Director of nursing	More than 200	100-499	No	No
I	Director of nursing	More than 200	100-499	No	No
J	Director of nursing	More than 200	100-499	No	No
K	Director of nursing	40-120	500-999	No	No
L	Nursing department staff	More than 200	100-499	No	No
M	Director of nursing	More than 200	100-499	No	No
N	Nursing department staff	More than 200	100-499	Yes	Yes
O	Director of nursing	More than 200	500-999	No	Yes

^※^ Period of time for counting the number of infected persons per 100,000 people by prefecture: Jan, 15, 2020-March 31, 2021 ^※※^The following answers in the interview Q1. Has the COVID-19 pandemic changed what hospitals are looking for in nurses? Q2. If the impact of the COVID-19 pandemic continues, will hospitals’ expectations of nurses change in the future?

The answers to the following two questions were tabulated during the interviews. Responses to the question, “Has the COVID-19 pandemic changed what hospitals are looking for in nurses?” were as follows: 3, yes; 11, no; and 1, unknown. Meanwhile, the three responses to the question, “If the impact of the COVID-19 pandemic continues, will hospitals’ expectations of nurses change in the future?” were as follows: 4, yes; 10, no; 1, do not know. Table 1 elaborates on these responses.

Furthermore, the qualities and reasons why hospitals require new graduate nurses because of the COVID-19 pandemic, extracted from verbatim records, were as follows: (1) things that have changed, which require the nurses’ ability to overcome challenges and desire for improvement; (2) things that may change in the future, in which the possibility of such changes requires nurses to have excellent communication skills and the ability to adapt to difficulties; and (3) changes are not necessary, the essence of nursing does not change in any situation, and incumbent education should be changed rather than hiring conditions. The results are presented in Table 2.

**Table 2.** Professional Qualities That Hospitals Require of New Graduate Nurses during the COVID-19 Pandemic.

Has the COVID-19 pandemic changed what hospitals are looking for in nurses?
*・Not only are they gentle and able to communicate, their strength and ability to overcome stress are also being tested. (E)*
*・They need to possess a clear sense of self-awareness, take pride in doing this job for the rest of their lives, and desire to improve themselves. (F)*
*・They have the ability to think for themselves immediately. (O)*
*・It is good to work with nurses who can look on the bright side while dealing with problems that may arise. (O)*
If the impact of the COVID-19 pandemic continues, will hospitals’ expectations of nurses change in the future?
*・Human resources are required to actively contribute to society and play an international role. (E)*
*・Human resources who can communicate clearly even in environments where they are wearing masks are required (G)*
*・Because I have not been in practical training, I have little experience talking to patients, and the nature of remote classes has weakened the camaraderie among students, so what I want is communication skills. (N)*
*・For difficult tasks, we need people with a sense of responsibility who can say that “We will take on the task,” rather than saying, “It cannot be helped because it is an order.” (O)*
*・We need nurses who can adapt to all kinds of difficulties. (O)*
The reason why I thought that COVID-19 did not change the humanity of nurses required by hospitals
*・Even if this situation continues, the essence of nursing will not change, and the quality required will not change because it is important to provide patient-centered care. (D)*
*・When I look back at new graduate nurses, I think they are doing enough nursing, and even those who are not good at communication have their own way of thinking, so I do not think there is such a big problem. (H)*
*・Rather than changing hiring conditions, I would like to collaborate with basic education teachers and reconsider the way of incumbent education. (K)*

Because of the COVID-19 pandemic, nurse managers expect certain changes in new graduate nurses’ qualities, including “their ability to overcome challenges and their desire for improvements.” The shortage of nurses has become a serious problem during the COVID-19 pandemic ^(4)^. As a policy-based approach by the government to address this issue, the need for strategies, such as appropriate distribution of support according to various risks of nursing work and clarification of support targets by screening nurses for mental health symptoms, has been suggested ^(5)^. In future university education programs, efforts to achieve adequate resilience will be required, particularly during learning opportunities close to the actual site, such as clinical training.

Because of the COVID-19 pandemic, “excellent communication skills” were cited as a change in the qualities that nurse managers may require from new graduate nurses in the future. The COVID-19 pandemic has limited individuals’ engagement and opportunities for face-to-face communication. Communication skills are an important part of nursing, a profession that necessitates interaction between nurses and patients. Verification of a training program using virtual humans is progressing toward addressing this issue ^(6)^, and a positive approach to communication skills is also required for nursing students.

Finally, approximately 25% of the nurse managers responded that the COVID-19 pandemic had changed the expectations of new graduate nurses. Among nurse managers seeking changes, there were no clear trends regarding job titles, hospital sizes, or pandemic impacts. Contrary to our expectation that many nurse managers would want a change in new nurses’ qualities, more than 70% answered that the qualities required of new graduate nurses would not change even after COVID-19. One reason was that “even if the pandemic continues, the essence of nursing will not change.” In other words, a fundamental attitude toward nursing is required, even during the COVID-19 pandemic. Additionally, the COVID-19 pandemic has affected the field of university education. Owing to the COVID-19 pandemic, the prevalence of depression and anxiety among college students has increased significantly ^(7)^, and nursing education has been forced to change from face-to-face to e-learning classes ^(8)^. These factors can influence the nurturing of the “attitudes toward nursing,” which will continue to be sought. Therefore, verifying the impact of the COVID-19 pandemic is necessary for nursing education outcomes to train new graduate nurses with the qualities nurse managers seek.

Despite providing certain insights, this study had several limitations. First, the effects of the COVID-19 pandemic are strongly influenced by national infection control measures and cultural background; hence, care must be taken when interpreting the results of this study. Second, some of the targeted medical institutions included areas where the spread of infections was noteworthy within Japan despite being limited to facilities located in certain areas. Data from these areas may not be generalizable to other areas in Japan. Therefore, further studies, including those that use quantitative methods, should be conducted in a larger number of target areas and facilities.

In conclusion, the qualities nurses required during the post-coronavirus pandemic period include excellent communication skills, self-awareness, pride in nursing, and the ability to overcome barriers. An educational system that nurtures these qualities must build a sustainable medical system.

## Article Information

### Conflicts of Interest

None

### Sources of Funding

This study was supported by the Promotion Project of The Next Generation Study, Post-Corona Field.

### Acknowledgement

We thank Editage (www.editage.jp) for English-language editing.

### Author Contributions

Michihiro Tsubaki: Conceptualization, Methodology, Validation, Formal analysis, Data curation, Writing - original draft, Visualization, Investigation, Writing - review & editing; Jun Kako: Conceptualization, Methodology, Validation, Investigation, Resources, Writing - review & editing, Funding acquisition, Supervision, Project administration; Yuji Koga: Conceptualization, Methodology, Validation, Investigation, Resources, Writing - review & editing, Supervision, Project administration; Masamitsu Kobayashi: Conceptualization, Methodology, Validation, Formal analysis, Data curation, Writing - original draft, Visualization, Investigation, Writing - review & editing, Supervision, Project administration; Yoji Endo: Conceptualization, Methodology, Writing - review & editing; Yasutaka Kimura: Conceptualization, Methodology, Validation, Investigation, Writing - review & editing; Hana Kiyohara: Conceptualization, Methodology, Investigation, Writing - review & editing; Michiko Ishida: Conceptualization, Methodology, Validation, Investigation, Writing - review & editing; Yoko Nishida: Conceptualization, Methodology, Validation, Investigation, Writing - review & editing; Kimie Harada: Conceptualization, Methodology, Investigation, Writing - review & editing; Kohei Kajiwara: Conceptualization, Methodology, Writing - review & editing; Yoshiyasu Ito: Conceptualization, Methodology, Investigation, Resources, Writing - review & editing; Yuki Wakiguchi: Conceptualization, Methodology, Writing - review & editing; Shinsuke Sasaki: Conceptualization, Methodology, Investigation, Resources, Writing - review & editing; Seiji Hamanishi: Conceptualization, Methodology, Writing - review & editing; Makoto Yamanaka Conceptualization, Methodology, Writing - review & editing; Takahiro Kakeda: Conceptualization, Methodology, Supervision, Project

### Approval by Institutional Review Board (IRB)

This study was approved by the Institutional Review Board of the Research Ethics Committee, College of Nursing Art & Science and Research Institute of Nursing Care for People and Community, University of Hyogo, Japan (approval no.: 2020F29, approval date: 3 March 2021).
